# Postprandial glucose and HbA1c are associated with severity of obstructive sleep apnoea in non-diabetic obese subjects

**DOI:** 10.1007/s40618-021-01602-8

**Published:** 2021-06-26

**Authors:** A. Cignarelli, A. Ciavarella, M. Barbaro, S. Kounaki, A. Di Trani, V. A. Falcone, V. N. Quaranta, A. Natalicchio, L. Laviola, O. Resta, F. Giorgino, S. Perrini

**Affiliations:** 1 Department of Emergency and Organ Transplantation - Section of Internal Medicine, Endocrinology, Andrology and Metabolic Diseases, University of Bari Aldo Moro, Bari, Italy; 2 Department of Basic Medical Sciences, Neurosciences and Sense Organs - Section of Respiratory Disease, University of Bari Aldo Moro, Bari, Italy

**Keywords:** Glycemia, Fasting, Postprandial, Obesity, Sleep apnea

## Abstract

**Introduction:**

Obstructive sleep apnoea (OSA) is an underdiagnosed condition frequently associated with glycaemic control impairment in patients with type 2 diabetes.

**Aim:**

To assess the relationship between glycometabolic parameters and OSA in obese non-diabetic subjects.

**Methods:**

Ninety consecutive subjects (mean age 44.9 ± 12 years, mean BMI 42.1 ± 9 kg/m^2^) underwent polysomnography and a 2-h oral glucose tolerance test (OGTT).

**Results:**

OSA was identified in 75% of subjects, with a higher prevalence of males compared to the group of subjects without OSA (62% vs 32%, *p* = 0.02). Patients with OSA had comparable BMI (42.8 kg/m^2^ vs 39.4 kg/m^2^), a higher average HbA1c (5.8% vs 5.4%, *p* < 0.001), plasma glucose at 120 min during OGTT (2 h-PG; 123 mg/dl vs 97 mg/dl, *p* = 0.009) and diastolic blood pressure (81.1 mmHg vs 76.2 mmHg, *p* = 0.046) than obese subjects without OSA. HbA1c and 2 h-PG were found to be correlated with the apnoea-hypopnoea index (AHI; *r* = 0.35 and *r* = 0.42, respectively) and with percent of sleep time with oxyhaemoglobin saturation < 90% (ST90; *r* = 0.44 and *r* = 0.39, respectively). Further, in a linear regression model, ST90 and AHI were found to be the main determinants of 2 h-PG (*β* = 0.81, *p* < 0.01 and *β* = 0.75, *p* = 0.02, respectively) after controlling for age, sex, waist circumference, physical activity, and C-reactive protein. Similarly, ST90 and AHI persisted as independent determinants of HbA1c (*β* = 0.01, *p* = 0.01 and *β* = 0.01, *p* = 0.01, respectively).

**Conclusion:**

Beyond the traditional clinical parameters, the presence of a normal-high value of 2 h-PG and HbA1c should raise suspicion of the presence of OSA in obese subjects.

## Introduction

Obstructive sleep apnoea (OSA) is a treatable chronic sleep-breathing disorder characterised by recurrent episodes of complete (apnoea) or partial (hypopnoea) obstruction of the upper airway resulting in intermittent hypoxia, arousals, and sleep fragmentation. Over the past decade, both pathophysiological and epidemiological studies have identified poor sleep quality and OSA as putative novel risk factors for type 2 diabetes (T2D) [1–5]. Moreover, a meta-analysis of five prospective studies reported that moderate-to-severe OSA confers a greater risk for T2D incidence [6] and, more recently, a significant improvement in glycaemic control and insulin resistance was demonstrated after treatment with continuous positive airway pressure (CPAP) in patients with type 2 diabetes and OSA [7]. Notably, OSA and disorders of glucose metabolism are both strongly associated with obesity and abdominal fat accumulation; thus, they not unexpectedly often occur concomitantly in the same individual. Indeed, several studies have established a robust association between the presence and severity of OSA and metabolic impairment in non-diabetic adults, independent of adiposity and other known confounders [3, 8–11]. Given that recurrent episodes of sleep-disordered breathing are followed by a cascade of events related to the activation of the sympatho-adrenal system, oxidative stress, systemic inflammation, and changes in adipokines involved in cardio-metabolic risk [12], the relationship between OSA and glucose homeostasis needs to be explored further. Thus, the purpose of this cross-sectional study was to investigate the association of OSA and various polysomnographic indexes with fasting glucose, postprandial glucose and HbA1c levels in high-risk non-diabetic patients with moderate/severe obesity.

## Methods

### Study design

This cross-sectional study included consecutive outpatients being screened for participation in a previously described randomised controlled trial (RCT) [12] to assess the effect of CPAP treatment on markers of inflammation in adipose tissue. The trial was conducted from February 2012 through December 2015 at the Outpatient Clinic for the Study of Obesity, Unit of Endocrinology, Department of Emergency and Organ Transplantation, University of Bari Aldo Moro. The trial protocol was approved by the Ethics Committee of the Azienda Ospedaliero-Universitaria Policlinico di Bari, Bari, Italy, and meets the standards of the 7th revision of the Declaration of Helsinki. Each subject provided written informed consent. The full details of the RCT have been detailed previously [12]. Briefly, inclusion criteria included recruitment at their first medical examination and BMI higher than 30 kg/m^2^. Exclusion criteria included known diabetes mellitus, plasma glucose diagnostic for diabetes [fasting glucose ≥ 126 mg/dl, 2 h post-oral glucose tolerance test (OGTT) ≥ 200 mg/dl, or both] or glycosylated hemoglobin (HbA1c) ≥ 6.5% [13], current smoking, autoimmune inflammatory diseases, cancer, severe kidney or liver diseases, stroke, ischemic or valvular heart disease, obesity hypoventilation syndrome, secondary causes of obesity (i.e., hypercortisolism, growth hormone deficiency), or use of medications that could affect body weight, glucose metabolism, and inflammatory markers, previous diagnosis of OSA and treatment for OSA. The present manuscript has followed the STROBE checklist guidelines [15].

### Anthropometric variables

Body weight and waist, hip and neck circumferences were measured. Blood pressure was measured to the nearest 2 mmHg using a periodically calibrated mercury sphygmomanometer in patients in the sitting position after at least 5 min of rest. The mean of three measurements was recorded. Physical activity was assessed using the International Physical Activity Questionnaire (IPAQ) [16]. The volume of both physical activity and time spent sitting per week were derived from the IPAQ validity and reliability study and expressed as (Metabolic Equivalent of Task (MET) × min/week and min/week, respectively) [16].

### Laboratory tests

Blood samples were drawn between 08:00 and 09:00 h after an overnight fast within 1 week of the ambulatory blood pressure measurement; all participants then underwent a standard 75-g OGTT. Serum insulin concentrations were measured by radioimmunoassay (Behring, Scoppitto, Italy). Plasma glucose levels before (FPG) and 2 h after (2 h-PG) OGTT were determined using the glucose-oxidase method (Sclavo, Siena, Italy), and plasma lipids [triglycerides, total cholesterol, and high-density lipoprotein (HDL) cholesterol] were measured using an automatic colorimetric method (Hitachi; Boehringer Mannheim, Mannheim, Germany). The low-density lipoprotein (LDL) cholesterol level was calculated using the Friedewald equation [17] and the estimated glomerular filtration rate (eGFR) level was calculated using the CKD-EPI equation [18]. Insulin sensitivity was estimated using the homeostasis model assessment method [19].

### Hypnological assessment

All patients underwent nocturnal cardiorespiratory monitoring with a portable cardiorespiratory monitor (SOMNEA) in ambient air and with spontaneous breathing for approximately 8 h within one month of their first outpatient check-up. The SOMNEA device consists of multiple sensors for the detection of the following signals: oxyhaemoglobin saturation (by a finger sensor), heart rate (derived from ECG electrodes placed on the chest), snoring sound (by a microphone placed on the thyroid cartilage), body posture, oro-nasal airflow (by a flow sensor for both nostrils and mouth) and thoracic and abdominal movements (by stretch belts). A respiratory event is defined as obstructive apnoea if it is characterised by a 90% reduction in airflow (compared to the mean of the previous 3 min) for at least 10 secs with preserved thoraco-abdominal movements. Obstructive hypopnoea is defined as a decrease in the airflow by 50% (compared to the mean of the previous 3 min) for at least 10 secs with preserved thoraco-abdominal movements associated with > 4% oxyhaemoglobin desaturation. Factors that were calculated include the number of obstructive apnoea/hypopnoea events per h of sleep [obstructive apnoea/hypopnoea index (AHI)], the number of oxyhaemoglobin desaturation > 4% events per h of sleep [oxyhemoglobin desaturation index (ODI)] and the time (expressed as a percentage of total actual sleep time) of oxyhaemoglobin saturation spent below 90% (ST90). All scoring was performed based on the American Academy of Sleep Medicine sleep scoring guidelines [20]. OSA diagnosis was proposed if a patient had an AHI index ≥ 5 with symptoms or an AHI index > 15 without symptoms [20].

### Arterial blood gas

Arterial blood gas was obtained in ambient air using radial artery puncture (arterial blood air-analyzer; Nova Biomedical Stat Profile Critical Care Xpress). The PaO_2_ and PaCO_2_ values were analysed.

### Statistical analysis

Continuous variables are presented as mean ± standard deviation (SD). Differences between patients with and without OSA were tested using Student’s *t* test or the Mann–Whitney *U* test for continuous variables according to normal distribution. Spearman’s correlation between HbA1c, FPG and 2 h-PG and hypnological variables was assessed. Categorical variables are reported as percentage and were compared using the *χ*^2^ test or Fisher’s exact test. All tests of significance were two-sided. Analysis was performed with RStudio for Windows, version 1.0.143. Normality was assessed using the Kolmogorov–Smirnov test. To estimate the effect size of increasing severity of OSA on FPG, 2 h-PG, and HbA1c in a clinically useful manner, the changes in FPG, 2 h-PG and HbA1c based on AHI and ST90 tertiles were statistically assessed with the non-parametric Kruskal–Wallis method. Linear regression analysis was applied to explore the relationships between glucose or HbA1c values and apnoea-related parameters independent of age, sex, waist circumference, physical activity or C-reactive protein (CRP). The covariates were included a priori in the model. The statistical significance level was set at 5% (*p* < 0.05). The post hoc analysis showed a power of 90% and *p* < 0.01 to establish an effect size of 1 for HbA1c, which represents a variation of 0.5%.

## Results

The anthropometric and metabolic characteristics of the 90 obese patients are presented in Table 1, according to the presence or absence of OSA. Sleep characteristics of the two groups are shown in Table 2. OSA was identified in 76% of patients, which were more frequently male as compared to patients without OSA (Non-OSA) (62% vs 38%, *p* = 0.02) (Table 1).

**Table 1:** Characteristics of the study population (*n* = 90)

Characteristic	Non-OSA	OSA	*p* value
*N* (%)	22 (24.5)	68 (75.5)	
Male sex (%)^‡^	7 (31.8)	42 (61.8)	**0.023**
Female sex (%)^‡^	13 (59.1)	26 (38.2)	
Age (years)	39.9 (13.7)	46.4 (10.4)	**0.020**
Current smoker (%)^‡^	4 (18.2)	16 (26.2)	0.388
BMI (kg/m^2^)	39.4 (8.0)	42.8 (9.7)	0.132
Neck circumference			
Female (cm)	39.1 (3.4)	40.5 (3.1)	0.211
Male (cm)	40.9 (1.9)	44.4 (3.1)	**0.002**
Height			
Female (cm)	158.9 (5.2)	158.9 (8)	0.993
Male (cm)	172.7 (3.9)	172.1 (7.5)	0.751
Weight (kg)			
Female (cm)	104.2 (19.4)	121.9 (20.5)	**< 0.001**
Male (cm)	104.4 (13.1)	118.6 (30.3)	0.051
Waist circumference			
Female (cm)	116.9 (12.3)	129.5 (20.1)	**0.019**
Male (cm)	116.3 (5.6)	126.8 (18)	**0.007**
Fasting glycaemia (mg/dl)	90.4 (13.8)	94.5 (10.7)	0.155
2 h-PG (mg/dl)	96.6 (18.0)	123.1 (32.3)	**0.009**
Fasting insulinemia (mUI/ml)	20.5 (13.0)	23.6 (11.9)	0.346
2 h-PI (mUI/ml)	94.6 (79.8)	86.3 (54.2)	0.687
HbA1c (%)	5.4 (0.5)	5.8 (0.5)	**< 0.001**
HOMA-IR	5.0 (3.3)	5.5 (2.8)	0.496
Systolic blood pressure (mmHg)	120.9 (16.0)	126.1 (12.7)	0.142
Diastolic blood pressure (mmHg)	76.2 (10.2)	81.1 (9.2)	**0.046**
HR (bpm)	73.1 (10.2)	75.4 (9.7)	0.372
Cholesterol			
Total (mg/dl)	178.6 (45.4)	190.3 (34.5)	0.209
LDL (mg/dl)	49.3 (12.9)	45.6 (12.5)	0.268
HDL (mg/dl)	105.8 (35.3)	119.3 (33.7)	0.130
Triglycerides (mg/dl)	118.9 (80.3)	126.1 (60.6)	0.273
Uric acid (mg/dl)	4.8 (1.2)	5.4 (1.4)	0.106
AST (UI/l)	26.6 (15.4)	23.3 (8.2)	0.264
ALT (UI/l)	48.5 (40.6)	41.5 (22.7)	0.379
γ-GT (UI/l)	37.1 (25.8)	37.1 (19.4)	0.994
MS (%)^‡^	5.0 (23.8)	21.0 (38.9)	0.336
MS score	2.0 (0.8)	2.3 (0.8)	0.348
CRP (mg/dl)	6.6 (7.1)	8.2 (7.2)	0.362
ESR (mean (sd))	17.4 (10.5)	23.7 (19.2)	0.174
WBC (*n* × 10^3^/ml)	6.6 (2.0)	7.7 (2.0)	**0.025**
Haematocrit (%)	41.0 (3.2)	42.8 (3.5)	0.071
Hb (g/dl)	14.0 (1.2)	14.3 (1.3)	0.434
METS (METS/week)^§^	9074 (21,872)	3779 (4459)	0.116
Sitting (min/week)^§^	407 (183)	393 (203)	0.825

Mean (SD): (unpaired Student’s *t* test). Bold font indicates *p* < 0.05

*2 h-PG* postprandial glucose 120 min after oral glucose tolerance test, *2 h-PI* postprandial insulin 120 min after oral glucose tolerance test, *ALT* alanine aminotransferase, *AST* aspartate aminotransferase, *BMI* body mass index, *CRP* C-reactive protein, *Hb* haemoglobin, *HbA1c* glycated haemoglobin A1c, *HDL* high-density lipoprotein, *HOMA-IR* homeostatic model assessment for insulin resistance, *HR* heart rate, *LDL* low-density lipoprotein, *METS* metabolic equivalent of task, *MS* metabolic syndrome, *SD* standard deviation, *WBC* white blood cells

^‡^Percentage (*χ*^2^ test for qualitative variable)

^§^Median; interquartile range in parentheses (Mann–Whitney *U* test)

**Table 2:** Polysomnographic and arterial blood gas variables in the study population

Characteristics	Non-OSA	OSA	*p* value
AHI (*n*/h)	4.10 (2.57)	33.77 (20.47)	**< 0.001**
ODI (*n*/h)	5.63 (3.44)	34.36 (20.47)	**< 0.001**
ST90 (%)	1.86 (2.64)	23.62 (21.93)	**< 0.001**
pH	7.43 (0.02)	7.43 (0.03)	0.633
PaO_2_ (mmHg)	89.12 (15.80)	78.20 (9.34)	**0.001**
PaCO_2_ (mmHg)	39.38 (2.45)	40.74 (3.08)	0.109
SO_2_ (%)	96.53 (1.55)	94.29 (9.30)	0.358
HCO_3_^−^ (mEq/l)	25.84 (1.07)	29.08 (9.67)	0.188
ESS	6.05 (2.74)	12.70 (3.00)	**< 0.001**
Mean oxygen saturation (%)	94.95 (1.25)	92.78 (3.17)	**0.002**
Sleep efficiency (%)	85.90 (11.06)	88.76 (8.81)	0.22
Supine position (%)	82.08 (22.59)	81.85 (25.16)	0.97

Mean ± SD (Unpaired Student’s *t* test). Bold font indicates *p* < 0.05

*AHI* apnoea-hypopnoea index, *ODI* oxygen desaturation index, *ESS* Epworth sleepiness score, *ST90* % of sleep time spent below 90% oxygen saturation, *SD* standard deviation

Additionally, the OSA group displayed older age (46.5 ± 10.4 years vs 39.9 ± 13.7 years, *p* = 0.02), higher diastolic blood pressure (81.1 vs 76.2 mmHg, *p* = 0.046), higher plasma glucose post-OGTT (2 h-PG; 123.1 vs 96.6 mg/dl, *p* = 0.01), and higher HbA1c (5.8% vs 5.4%, *p* < 0.001). Moreover, BMI did not differ between the OSA and non-OSA groups (*p* = 0.10), whereas both male and female patients with OSA had a higher average waist circumference (male: 126.8 vs 116.3 cm, *p* = 0.01; female: 129.5 vs 116.9, *p* = 0.02), and male patients with OSA had a higher average neck circumference than non-OSA patients (44.4 vs 40.9 cm, *p* = 0.002; Table 1). However, the prevalence of metabolic syndrome did not differ between the two groups (*p* = 0.34; Table 1). Interestingly, OSA patients displayed higher white blood cells (7.7 vs 6.6 *n* × 10^3^/mm^3^, *p* = 0.02; Table 1).

Results of the Spearman correlation showed a significant moderate and positive association between apnoic/hypopnoic events and nocturnal hypoxemia and glycemia. Particularly, both 2 h-PG and HbA1c appear to be correlated with AHI (*r* = 0.42, *p* < 0.01 and *r* = 0.35, *p* < 0.001, respectively). Likewise, a significant moderate and positive association between both 2 h-PG and HbA1c and ST90 was observed (*r* = 0.39, *p* < 0.01 and *r* = 0.44, *p* < 0.001, respectively; Table 3). In contrast, FPG appeared to be not correlated to either AHI or ST90. Notably, uric acid was correlated with AHI (*r* = 0.27, *p* = 0.02) and ST90 (*r* = 0.40, *p* < 0.001; data not shown).

**Table 3:** Spearman correlation matrix for continuous variable

	FPG	2 h-PG	HbA1c	Waist	BMI	Neck	AHI	ESS
FPG								
2 h-PG	0.07							
HbA1c	0.29**	0.42**						
Waist	0.07	0.12	0.32**					
BMI	0.04	0.19	0.32**	0.79***				
Neck	0.33**	0.29*	0.14	0.41***	0.18			
AHI	0.15	0.42**	0.35***	0.34**	0.16	0.4***		
ESS	0.13	0.15	0.32**	0.28*	0.23*	0.25*	0.45***	
ST90	0.2	0.39**	0.44***	0.46***	0.32**	0.4***	0.79***	0.46***

*FPG* fasting plasma glucose, *2 h-PG* postprandial glucose 2 h after oral glucose tolerance test, *HbA1c* glycated haemoglobin A1c, *BMI* body mass index, *AHI* apnoea-hypopnoea index, *ESS* Epworth sleepiness scale, *ST90* % of sleep time spent below 90% oxygen saturation

*p < 0.05, **p < 0.01, ***p < 0.001

**Fig. 1:**
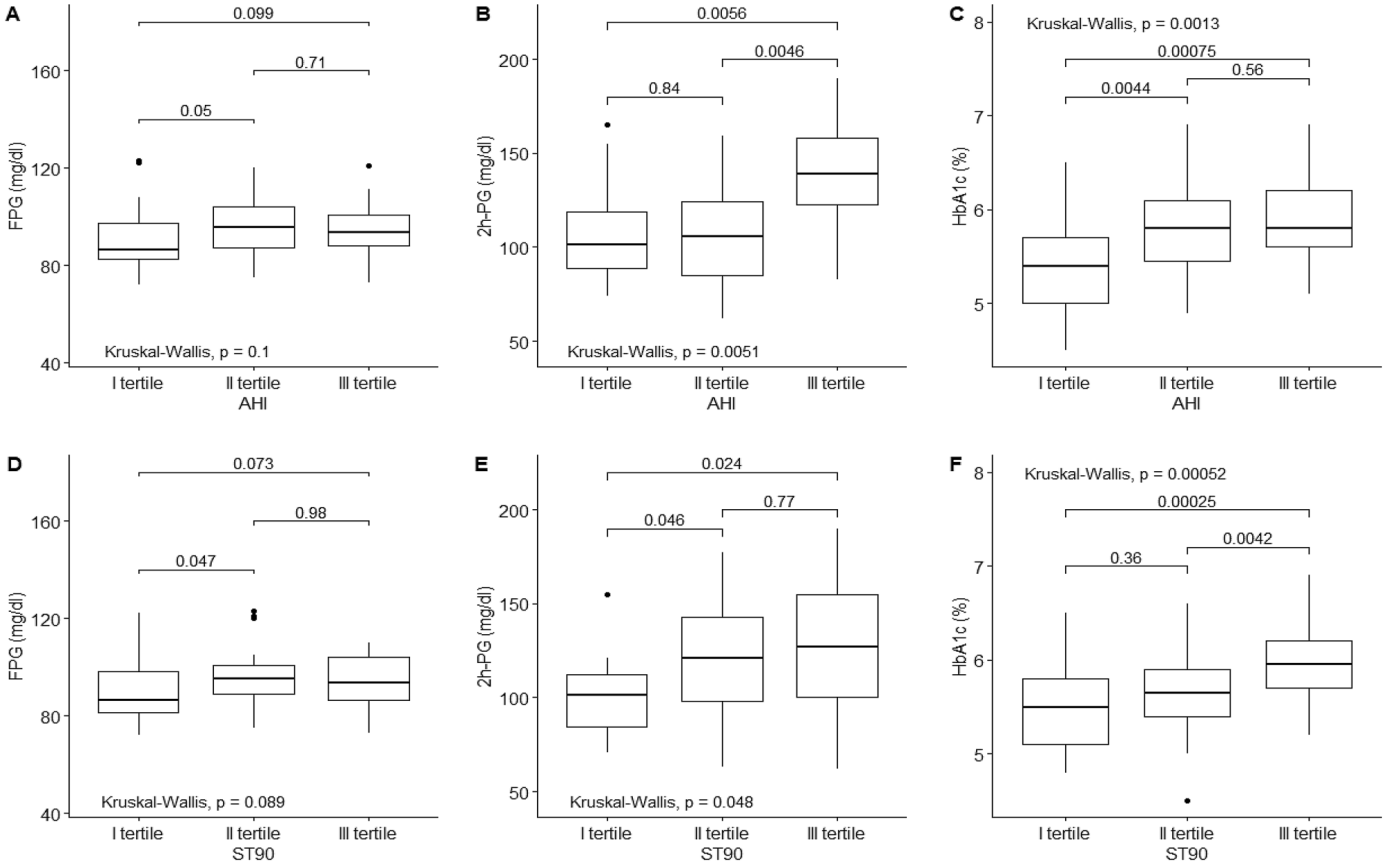
Glycaemic parameters according to tertiles of apnoeic indexes. **A**–**C** FPG, 2 h-PG and HbA1c boxplots according to AHI tertiles, respectively. **D**–**F** FPG, 2 hPG and HbA1c boxplots according to ST90 tertiles, respectively. Abbreviations: *AHI* Apnoea-hypopnoea index, *ST90* % of sleep time spent below 90% oxygen saturation, *FPG* Fasting plasma glucose, *2 h-PG* Postprandial glucose 2 hours after oral glucose tolerance test, *HbA1c* Glycated haemoglobin A1c

Next, ST90 and AHI variables were categorised into tertiles. Higher ST90 and AHI tertiles were associated with significantly higher levels of HbA1c (+ 0.5%, + 0.4%, tertile III vs I, respectively; *p* < 0.05) and 2 h-PG (+ 24 mg/dl, + 32 mg/dl, tertile III vs I, respectively; *p* < 0.05; Fig. 1).

A multivariate regression model in which FPG, 2 h-PG, and HbA1c were considered as dependent variables was constructed using AHI or ST90 as independent variables and age, sex, waist circumference, physical activity and CRP as covariates. Both AHI and ST90 were significant associated with higher HbA1c and 2 h-PG (Table 4), but not with FPG. The model indicates that each increase of 13 *n*/h in AHI (*p* = 0.02) and of 12% in ST90 (*p* = 0.003) is associated with a 10 mg/dl increase in 2 h-PG. Moreover, an increase of 9 *n*/h in AHI (*p* = 0.01) and of 9% in ST90 (*p* = 0.01) was associated with an increase of 1 mmol/mol in HbA1c (Table 4).

**Table 4:** Regression table

Dependent variable	*β*	SE	T-statistic	*p* value
AHI				
FPG	0.136	0.096	1.411	0.170
2 h-PG	0.749	0.312	2.404	**0.022**
HbA1c	0.011	0.004	2.655	**0.011**
ST90				
FPG	0.009	0.093	0.101	0.920
2 h-PG	0.815	0.259	3.142	**0.003**
HbA1c	0.010	0.004	2.540	**0.015**

Bold font indicates *p* < 0.05

*AHI* apnoea-hypopnoea index, *ST90* % of sleep time spent below 90% oxygen saturation, *FPG* fasting plasma glucose, *2 h-PG* postprandial glucose 2 h after oral glucose tolerance test, *HbA1c* glycated haemoglobin A1c, *SE* standard error

## Discussion

This study examined the potential correlation between the sleep-breathing disorder OSA and glycaemic parameters in non-diabetic obese patients. Although obesity is the main risk factor for the development of T2D, coexisting OSA may add to this risk. We found greater than 75% prevalence of OSA in individuals with obesity, which is consistent with previous reports [20–23]. Sleep-breathing disorders may influence glucose and HbA1c levels independent of central obesity [23–26]. A recent meta-analysis of prospective cohort studies suggests that moderate-severe OSA may increase the risk of T2D (RR 1.63; 95% confidence interval 1.09–2.45), supporting the hypothesis that OSA may represent an independent risk factor for the development of this disease [6]. A significant correlation between nocturnal hypoxemia and HbA1c was found in other studies on non-diabetic individuals [24, 27, 28]. Additionally, the presence of OSA may lead to a higher glycaemic variability, defined as mean amplitude of glycaemic excursion (MAGE) [29]; in fact, an association between MAGE and AHI has been confirmed in non-diabetic patients as well [30]. However, the relationship between OSA and glycaemic parameters in morbidly obese individuals without diabetes is poorly defined. In this study, we show a significant correlation of HbA1c and 2 h-PG but not FPG with AHI and ST90. Moreover, multiple regression analysis indicated that ST90 and AHI display the highest *b* value and thus represent the main determinants of 2 h-PG and HbA1c among the multiple potentially confounding factors (data not shown).

Therefore, metabolic abnormalities found in morbid obesity may be linked not only to adiposity per se but also to concomitant OSA. Several mechanisms, including direct effects of hypoxia [31], oxidative stress [32, 33] sympathetic nervous system activation [34] and the associated increase in catecholamines [34–37], and alterations in pro-inflammatory cytokines such as interleukin-6, tumor necrosis factor-*a* [38] and hypoxia-inducible factor-1*a* [39] seem to play a critical role in the metabolism of carbohydrates. In a recent study investigating the effects of 24 weeks of weight-loss intervention plus CPAP therapy in obese individuals with OSA, we have shown that correction of intermittent hypoxemia improves systemic and obesity-associated inflammatory markers [12]. In OSA, increased sympathetic nerve activity and catecholamines may occur as a result of repeated arousals or repeated oxygen desaturations [40]. In humans, exposure to high altitude hypoxia for a few days increases glucose and insulin concentrations together with increased plasma catecholamines and cortisol [36, 41, 42]. Moreover, OSA is reportedly associated with marked impairments in insulin sensitivity and disposition index (an integrated measure of pancreatic ß-cell function) independent of adiposity [9, 43]. Finally, intermittent hypoxemia has been shown to be toxic to ß-cell function in murine models of sleep apnoea [44, 45].

Our data suggest how OSA may worsen HbA1c and postprandial glycaemia, in particular, in individuals with morbid obesity before T2D develops. In this study, a significant correlation between nocturnal hypoxemia or apnoea index with FPG was not found, even though the lowest tertile of AHI and ST90 were found to be associated with lower FPG compared to the other tertiles. However, in a non-T2D population, a study on 31 male individuals found a significant correlation between minimum O_2_ level, but not AHI, with FPG [27]. This small discrepancy could be due to differences in the study populations, since individuals with substantially lower BMIs were analysed in that study. It could be argued that the effects of OSA on FPG are more prominent in leaner individuals, while in higher-grade obesity the influence of hypoxemia injury on ß-cell dysfunction and metabolic abnormalities could be more pronounced. Although this study was not designed to examine the mechanisms for the adverse effect of OSA on glycaemic parameters, the results support the hypothesis that OSA-induced intermittent hypoxia could exert harmful effects on glucose metabolism primarily by increasing 2 h-PG. Our results are in accordance with the findings of Babu et al., who demonstrated that CPAP treatment results in a significant reduction of 1-h postprandial glucose in diabetic patients with OSA [46].

Both postprandial glucose and HbA1c exhibited a significant correlation with nocturnal hypoxemia indexes, but not with sleep fragmentation. This finding suggests that the effect of OSA on glucose parameters may be mediated by hypoxia and not by sleep alterations. However, Grimaldi et al. showed that HbA1c levels are associated with obstructive apnoeas that occur particularly during REM sleep, suggesting the relevance of REM sleep integrity for glucose control [47]. Nevertheless, sleepiness as a surrogate marker of sleep integrity seems to be associated with glucose control only in subjects affected by a low grade of obesity [48], and in this study, using sleep efficiency in the multivariate model did not change the results of the regression analysis (data not shown).

Interestingly, even if only a few patients showed hyperuricaemia, plasma uric acid displayed a positive correlation with AHI and ST90, suggesting that OSA is a potential risk factor for the development of hyperuricaemia. Of note, we have recently demonstrated that CPAP treatment could reduce uric acid levels in OSA patients [12]. Further studies are required to address whether higher plasma uric acid levels, as a consequence of more severe forms of OSA, may directly affect glucose metabolism and/or cardiovascular risk in these individuals.

Moreover, several other blood metabolites other than 2 h-PG, HbA1c and uric acid, such as circulating complement component 3, CRP and erythropoietin, have recently been raised as promising biomarkers supporting the diagnosis of OSA [48–51].

In conclusion, OSA represents common comorbidity among non-diabetic morbidly obese patients. In this population, the presence of normal-high levels of either 2 h-PG or HbA1c may help in identifying the presence of OSA, which acts as an independent marker of a peculiar metabolic derangement in obese patients. Indeed, OSA is associated with higher postprandial glycaemia and HbA1c even in normoglycaemic patients and this occurs independently of gender, age and central obesity. Recognition of OSA may thus substantiate aggressive treatment of obesity and intermittent hypoxia to antagonise the progression to T2D in obese patients.

## Data Availability

Data are available for reviewer check.
